# Induction therapy with ipilimumab and nivolumab followed by consolidative chemoradiation as organ-sparing treatment in urothelial bladder cancer: study protocol of the INDIBLADE trial

**DOI:** 10.3389/fonc.2023.1246603

**Published:** 2023-08-29

**Authors:** C.F. Stockem, J.J.J. Mellema, B.W.G. van Rhijn, T.N. Boellaard, M.L. van Montfoort, S. Balduzzi, J.L. Boormans, M. Franckena, R.P. Meijer, D.G.J. Robbrecht, B.B.M. Suelmann, E.E. Schaake, M.S. van der Heijden

**Affiliations:** ^1^ Department of Medical Oncology, Netherlands Cancer Institute, Amsterdam, Netherlands; ^2^ Department of Oncological Urology, Netherlands Cancer Institute, Amsterdam, Netherlands; ^3^ Department of Radiology, Netherlands Cancer Institute, Amsterdam, Netherlands; ^4^ Department of Pathology, Netherlands Cancer Institute, Amsterdam, Netherlands; ^5^ Department of Statistics, Netherlands Cancer Institute, Amsterdam, Netherlands; ^6^ Department of Oncological Urology, Erasmus Medical Center, Rotterdam, Netherlands; ^7^ Department of Radiotherapy, Erasmus Medical Center, Rotterdam, Netherlands; ^8^ Department of Oncological Urology, University Medical Center (UMC), Utrecht, Netherlands; ^9^ Department of Medical Oncology, Erasmus Medical Center, Rotterdam, Netherlands; ^10^ Department of Medical Oncology, University Medical Center (UMC), Utrecht, Netherlands; ^11^ Department of Radiation Oncology, Netherlands Cancer Institute, Amsterdam, Netherlands

**Keywords:** Muscle-invasive bladder cancer, bladder preservation, immune checkpoint blockade, chemoradiation, trimodal therapy

## Abstract

**Introduction:**

Studies that assessed the efficacy of pre-operative immune checkpoint blockade (ICB) in locally advanced urothelial cancer of the bladder showed encouraging pathological complete response rates, suggesting that a bladder-sparing approach may be a viable option in a subset of patients. Chemoradiation is an alternative for radical cystectomy with similar oncological outcomes, but is still mainly used in selected patients with organ-confined tumors or patients ineligible to undergo radical cystectomy. We propose to sequentially administer ICB and chemoradiation to patients with (locally advanced) muscle-invasive bladder cancer.

**Methods:**

The INDIBLADE trial is an investigator-initiated, single-arm, multicenter phase 2 trial. Fifty patients with cT2-4aN0-2M0 urothelial bladder cancer will be treated with ipilimumab 3 mg/kg on day 1, ipilimumab 3 mg/kg plus nivolumab 1 mg/kg on day 22, and nivolumab 3 mg/kg on day 43 followed by chemoradiation. The primary endpoint is the bladder-intact event-free survival (BI-EFS). Events include: local or distant recurrence, salvage cystectomy, death and switch to platinum-based chemotherapy. We will also evaluate the potential of multiparametric magnetic resonance imaging of the bladder to identify non-responders, and we will assess the clearance of circulating tumor DNA as a biomarker for ICB treatment response.

**Discussion:**

This is the first trial in which the efficacy of induction combination ICB followed by chemoradiation is being evaluated to provide bladder-preservation in patients with (locally advanced) urothelial bladder cancer.

**Clinical Trial Registration:**

The INDIBLADE trial was registered on clinicaltrials.gov on January 21, 2022 (NCT05200988).

## 1 Introduction

Bladder cancer is the tenth most common malignancy worldwide. Although mostly non-invasive at diagnosis, approximately 25% of the patients has a more aggressive bladder tumor, which involves the muscle-layer surrounding the bladder (1). The standard treatment of muscle-invasive bladder cancer (MIBC) usually involves radical cystectomy. However, five year overall survival (OS) ranges from 77% for pT2N0 patients to 44% for pT4aN0 patients after radical cystectomy, whereas five year OS is only 31% in case of node-positive disease (2). To improve outcomes following radical cystectomy, neo-adjuvant platinum-based chemotherapy is integrated in clinical guidelines since 1980, resulting in an ypT0N0 and ≤ypT1N0 at radical cystectomy in 22.7% and 40.8% of the cases respectively (3). Even though these response rates are encouraging, the absolute benefit of neo-adjuvant chemotherapy in terms of OS is only around 5% (4).

Radical cystectomy is associated with an increased risk of both morbidity and mortality. This is reflected by a complication rate of 58.5% and a mortality rate of 4.7% within 90 days after radical cystectomy (5). An alternative for radical cystectomy in patients with organ-confined disease or in patients who are considered unfit for radical cystectomy is trimodal therapy (TMT), which includes a transurethral resection of the bladder tumor (TUR-B), chemotherapy (consisting of mitomycine plus fluoropyrimidines, cisplatin or gemcitabine) and concurrent radiation. A randomized controlled trial to assess feasibility of comparing radical cystectomy with TMT demonstrated that a one-to-one comparison of the two treatment modalities is not feasible (6). Nevertheless, it has been shown that the combination of chemotherapy and radiation leads to similar survival outcomes when indirectly compared to radical cystectomy (7) and to achieve superior local control when compared to external beam radiation alone as a bladder-sparing approach (8).

To improve outcomes in patients with urothelial bladder cancer, immune checkpoint blockade (ICB) has been studied intensively in the last decade. Pembrolizumab, an antibody targeting programmed cell death protein 1 (PD-1), resulted in superior OS in comparison to second-line chemotherapy (paclitaxel, docetaxel or vinflunine) in metastatic urothelial cancer (9). Maintenance therapy with avelumab, targeting the PD-1 ligand PD-L1, is recommended in current clinical guidelines for patients who have at least stable disease following first line platinum-based chemotherapy (10). Recently, based on results of the CheckMate 274 demonstrating a benefit in disease-free survival after adjuvant nivolumab compared to placebo (22.0 months vs 10.9 months; HR 0.71), adjuvant nivolumab is approved by the Food and Drug Administration for patients with pT3-pT4a or pN+ disease who were not treated with neo-adjuvant cisplatin-based chemotherapy, and for patients with ypT2-ypT4a or yN+ disease who were treated with neo-adjuvant cisplatin-based chemotherapy (11). In Europe, adjuvant nivolumab is only approved for patients with muscle-invasive bladder cancer who have a PD-L1 expression on tumor cells of ≥1%. Despite the fact that anti-PD-(L)1 is integrated in current clinical guidelines, a majority of the patients treated with single-agent ICB is unresponsive (9, 10, 12–19). Reasons for this resistance may include inadequate priming of the immune system by cancer antigens and/or negative regulation of other steps in the cancer immunity cycle (20). In order to overcome ineffectiveness of single-agent ICB, targeting other inhibitory immune checkpoints has been the subject of extensive research. CTLA-4 is another inhibitory immune checkpoint on activated T-cells, and its inhibition may prevent negative regulation of T-cell priming, thereby broadening the immune response to cancer antigens. In patients with stage IV urothelial cancer who experienced progressive disease during or after platinum-based chemotherapy, combination ICB with nivolumab (anti-PD-1) and ipilimumab (anti-CTLA-4) led to improved response compared to nivolumab alone (21). In the phase 3 DANUBE trial, the combination of durvalumab (anti-PD-L1) and tremelimumab (anti-CTLA-4) was compared to standard of care chemotherapy as frontline treatment in stage IV urothelial cancer. Median OS was significantly longer following combination ICB (17.9 months) versus chemotherapy (12.1 months; hazard ratio 0.74) in PD-L1 positive patients, but as the primary endpoint involved OS in the intention-to-treat population, this trial was considered negative (19). Nevertheless, results of the DANUBE trial suggest that there could be enhanced anti-tumour effects of combination ICB compared to monotherapy in a subgroup of patients.

Combination ICB has been studied in earlier stages of urothelial cancer as well. The rationale for this strategy is based on encouraging results of combination ICB in other resectable malignancies (22–25). In the first cohort of the phase 1b NABUCCO trial, 24 patients with locally advanced urothelial cancer were treated with three subsequent cycles of ipilimumab 3 mg/kg and nivolumab 1 mg/kg pre-operatively to assess feasibility to undergo radical cystectomy within 12 weeks from treatment start (26). The primary endpoint was met, as there was only one patient who underwent cystectomy later than 12 weeks after study start due to immune-related toxicity. Grade 3-4 immune-related adverse events (irAE) were observed in 55% of the patients and in 41% of the patients when excluding laboratory abnormalities without clinical relevance. As pre-operative combination ICB was considered feasible based on results of the first NABUCCO cohort, 30 additional patients were randomized to ipilimumab “high” (3 mg/kg) plus nivolumab or ipilimumab “low” (1 mg/kg) plus nivolumab in a second cohort (27). Despite ≥grade 3 irAE in 33% of the patients treated with ipilimumab 3 mg/kg versus 20% in the ipilimumab 1 mg/kg arm, both treatment regimens were considered feasible. As efficacy in terms of pathological complete response (pCR, pT0N0) was achieved in 46% in cohort 1 (ipi 3 mg/kg) and in 43% of the “ipi high” arm of cohort 2 versus 7% in the “ipi low” arm, ipilimumab in a dose of 3 mg/kg was considered necessary to achieve an effective anti-tumor response in urothelial cancer (26, 27). In addition, in both NABUCCO cohorts, absence of plasma ctDNA pre-surgery was highly correlated with pCR (odds ratio=45.0; CI=4.9-416.5; p<0.01) and with PFS (hazard ratio =10.4; CI=2.9-37.5; p<0.001) (27).

Based on the promising anti-tumor effects with respect to pCR and clinical outcome upon combination ICB observed in the NABUCCO trial (26–28), we argue that TMT may be feasible in patients with (locally advanced) urothelial cancer after induction treatment with ICB. Despite recommendations for TMT in a selected group of patients, only 7.6% of the MIBC patients in North America is treated with (chemo)radiation (29). In The Netherlands, 301 out of 2657 patients with cT2-4aN0-2M0 urothelial bladder cancer were treated with chemoradiation based on a nationwide cohort study (30). As neo-adjuvant chemotherapy has been demonstrated to result in a pCR in 38% (31, 32), some trials consider this subset of patients appropriate for bladder preservation without local treatment of the bladder, thereby sparing patients from potential treatment associated toxicity. However, as the risk for disease recurrence is high in patients with cT2-T4a bladder tumors, we expect that systemic treatment alone is insufficient to result in long-term disease control in the majority of patients. In the randomized BC2001 trial, cumulative toxicity of grade≥3 was demonstrated in 9.2% of patients treated with chemoradiation versus 17% in patients treated with irradiation alone (33). As radiation techniques have improved since the treatment period in this trial, radiation-related toxicity is expected to be even lower in current radiation treatment schedules. We thus believe that a higher rate of bladder preservation has preference over avoiding radiation-related toxicity, especially in high-risk patients.

As we hypothesize that combination ICB followed by chemoradiation could be an effective bladder-sparing approach in patients with (locally advanced) urothelial cancer of the bladder, we initiated the INDIBLADE trial. We aim to enroll 50 patients for induction treatment with ipilimumab plus nivolumab followed by consolidative chemoradiation to assess bladder-intact event-free survival (BI-EFS).

The Pure-01 study showed that multiparametric magnetic resonance imaging (mpMRI) of the bladder was predictive for pCR following neo-adjuvant pembrolizumab (34). In the INDIBLADE trial, we will also evaluate the potential of mpMRI to distinguish responders from non-responders, aiming to identify patients in whom bladder preservation is feasible.

To enable personalized medicine, liquid biopsies could be used as biomarkers to evaluate response to treatment and potentially de-intensify therapy. As the absence of plasma ctDNA after neo-adjuvant ICB was associated with treatment response in NABUCCO (27), we aim to study whether we can use clearance of ctDNA to select patients for bladder-sparing treatment.

## 2 Methods and analysis

### 2.1 Study design

The INDIBLADE trial is an investigator-initiated, multicenter, single-arm, open label phase 2 trial investigating the efficacy of induction therapy with ICB followed by consolidative chemoradiation to spare the bladder in patients with (locally advanced) muscle-invasive urothelial bladder cancer. Participating sited are the Netherlands Cancer Institute (NKI; Amsterdam, NL), University Medical Center Utrecht (UMCU; Utrecht, NL), and Erasmus Medical Center (Rotterdam, NL).

### 2.2 Study population

Adult patients with cT2-4aN0-2M0 urothelial bladder cancer who are considered fit for chemoradiation are eligible for enrollment. We recognize that patients with nodal disease have a high risk for recurrence and may not traditionally be viewed as patients where cystectomy and pelvic lymph node dissection can be omitted. However, these patients are mainly at risk for distant recurrence. We believe that efficacy of systemic induction therapy with ICB, as was observed in node positive patients in NABUCCO (26, 27), could make node-positive patients eligible for a bladder-sparing approach. Pelvic lymph nodes suspected of metastasis at baseline will be included in the radiation field. A complete overview of all in- and exclusion criteria is provided in **Table 1**.

**Table 1:** In- and exclusion criteria for participating in the INDIBLADE trial.

Inclusion criteria	Exclusion criteria
- Willing and able to provide informed consent - Age ≥ 18 yearst - Patients with cT2-4aN0-2M0 urothelial bladder cancer - Lymph nodes should be amenable for inclusion into the radiation field - WHO performance Status 0 or 1 - Urothelial cancer is the dominant histology (>70%)^1^ - Availability of FFPE tumor specimens in paraffin blocks from diagnostic TUR - Screening laboratory values must meet the following criteria: WBC ≥ 2.0x109/L, Neutrophils ≥1.0x109/L, Platelets ≥100 x109/L, Hemoglobin ≥5.5 mmol/L, GFR>30 ml/min as per Cockcroft-Gault formula, AST ≤ 2.5 x ULN, ALT ≤2.5 x ULN, Bilirubin ≤1.5 X ULN - Negative pregnancy test (βHCG in urine or blood) within 2 weeks prior to day 1 of start immunotherapy for WOCBP - Highly effective contraception for both male and female subjects if the risk of conception exists; WOCBP must comply with contraception methods as requested by the study protocol	- Prior pelvic irradiation - UTUC - Extensive CIS of the bladder - Contra-indication to one of the study treatment components, or mpMRI - Subjects with active autoimmune disease in the past 2 years^2^ - Documented history of severe autoimmune disease (e.g. inflammatory bowel disease, myasthenia gravis) - Prior CTLA-4 or PD-(L)1 -targeting immunotherapy - Known history HIV, active TBC, or other active infection requiring therapy at the time of inclusion - Positive tests for HBsAg or HCV RNA - Underlying medical conditions that, in the investigator’s opinion, will make the administration of study drug hazardous or obscure the interpretation of adverse events - Medical condition requiring the use of immunosuppressive medications^3^ - Use of other investigational drugs four weeks or five half lives before study drug administration - Malignancy, other than urothelial cancer, in the previous 2 years, with a high chance of recurrence (estimated >10%)^4^ - Pregnant and lactating female patients - Major pelvic surgical procedure within 4 weeks prior to enrolment or anticipation of need for a major surgical procedure during the course of the study other than for diagnosis - Severe infections within 2 weeks prior to enrolment in the study including but not limited to hospitalization for complications of infection, bacteremia, or severe pneumonia

^1^ A small cell component is not allowed.

^2^ Patients with diabetes mellitus, properly controlled hypothyroidism or hyperthyroidism, vitiligo, psoriasis or other mild skin disease can still be included.

^3^ With the exceptions of intranasal and inhaled corticosteroids or systemic corticosteroids at physiological doses, which are not to exceed 10 mg/day of prednisone, or an equivalent corticosteroid. Steroids as premedication for hypersensitivity reactions (e.g., CT scan premedication) will be allowed.

^4^ Patients with low risk prostate cancer (defined as Stage T1/T2a, Gleason score ≤ 6, and PSA ≤ 10 ng/mL) who are treatment-naive and undergoing active surveillance are eligible.

WHO, world health organization; FFPE, Formalin-fixed paraffin-embedded; TUR, transurethral resection; WBC, white blood cell count; GFR, glomerular filtration rate; AST, aspartate aminotransferase; ALT, alanine transaminase; ULN, upper limit of normal; WOCBP, women of childbearing potential; UTUC, upper tract urothelial cancer; CIS, carcinoma in situ; IV, intravenous; mpMRI, multiparametric magnetic resonance imaging; HIV, Human Immunodeficiency Virus; TBC, tuberculosis; HBsAg, Hepatitis B surface antigen; HCV RNA, Hepatitis C ribonucleic acid; bHCG, beta-subunit of human chrorionic gonadotropin.

### 2.3 Study procedures and interventions

#### 2.3.1 ICB regimen

**Figure 1:**
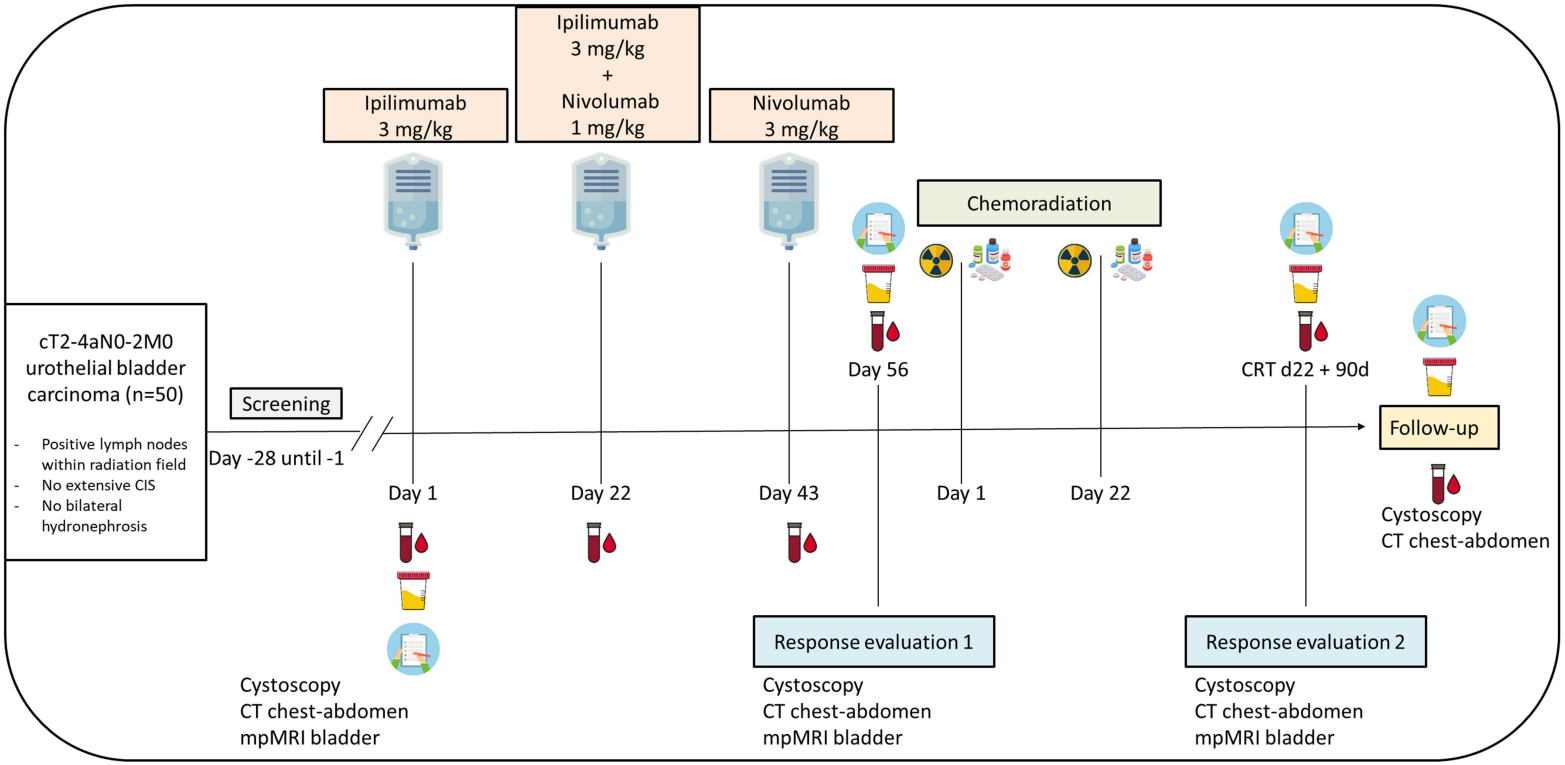
Study scheme INDIBLADE trial. Plasma will be drawn for ctDNA analysis before each cycle with immune checkpoint blockade, at each response evaluation, and during follow-up until 12 months after completing chemoradiation. Urine will be collected for ctDNA detection at baseline, at each response evaluation and during follow-up until 12 months after completing chemoradiation. EORTC QLQ-C30 and -BLM30 questionnaires will be collected at baseline, at each response evaluation, and during follow-up until 12 months after completing chemoradiation.

Eligible patients will undergo study treatment which consists of ipilimumab 3 mg/kg on day 1, ipilimumab 3 mg/kg plus nivolumab 1 mg/kg on day 22, and nivolumab 3 mg/kg on day 43 (**Figure 1**). This treatment schedule is based on the comparison of different dosing regimens in the NABUCCO trial, suggesting that ipilimumab in a high dosage (3 mg/kg) is most effective in urothelial cancer (27). We choose to administer ipilimumab alone in the first treatment cycle as was done in the first cohort of NABUCCO, because PFS appeared to be better when ipilimumab was administered alone rather than in combination with nivolumab in the first cycle (27). As we aim to specifically evaluate the efficacy of induction therapy with combination ICB, we do not allow other neo-adjuvant/induction agents, such as cisplatin-based chemotherapy.

#### 2.3.2 Radio sensitizing chemotherapy

Both the European and the American Urology Association recommend either cisplatin or mitomycin C (MMC) combined with 5-Fluorouracil (5-FU) as radio sensitizing chemotherapeutic agent, as these two regimens have been studied most extensively (35, 36). To our knowledge, these agents have not been compared in urothelial bladder cancer. As there is level 1 evidence for the combination of MMC plus fluoropyrimidines and to minimize the variation in treatment regimens, we chose MMC plus fluoropyrimidines as the radiosensitising regimen in this trial. Capecitabin, an oral prodrug of 5-FU, could be an alternative for 5-FU, as it is orally available and therefore does not require a continuous IV infusion for an extended period. In phase 3 trials in gastro-intestinal cancer, capecitabin was shown to have similar efficacy compared to 5-FU (37–39). A retrospective study performed at our own institution in urothelial cancer demonstrated that capecitabin is an alternative for 5-FU as radio sensitizer during chemoradiation, with comparable short-term disease-free survival rates compared to MMC/5-FU plus radiotherapy (40). In addition, capecitabin and 5-FU have recently been shown to have comparable OS and PFS at two years in a Dutch nationwide cohort study (30). MMC followed by capecitabin has become common practice in the Netherlands. In the current trial, patients will receive MMC 12 mg/m2 intravenously (with a maximum dose of 20 mg) on the first day of radiotherapy. Sensitizing chemotherapy is preferably oral capecitabin 825 mg/m2 twice daily on the same days as radiotherapy is given. If capecitabin is contraindicated, 5-FU can be given intravenously daily on days 1-5 and 22-26 of radiotherapy. In case of a deficiency for dihydropyrimidine dehydrogenase, dosing of capecitabin or 5-FU may be adjusted.

#### 2.3.3 Radiotherapy

Radiation treatment will be administered during four to six weeks (according to institutional protocol) using intensity modulated radiation therapy. Fifty-five gray or a bioequivalent dose covering ≥95% of the total bladder as clinical target volume and visible tumor for focal boosting should be applied. Solitary tumors can be irradiated with a partial boost after lipiodol injections, bladder fiducials [e.g. BioXmark (41)] or MRI guidance during treatment. Both CT- and MR-guided linac can be used to administer radiotherapy. More detailed information regarding interventions and assessments during this trial is depicted in **Table 2**.

**Table 2:** Schedule of interventions and assessments.

		Induction therapy				Chemoradiation			
Intervention/assessment	Screening	Ipi 3 mg/kg	Ipi 3 mg/kg nivo 1 mg/kg	Nivo 3 mg/kg	Response evaluation 1	Day 1^1^	Day 22	Response evaluation 2	Follow-up^2^
Timeline	Days -28 till -1	Day 1 ( ± 3 days)	Day 22 ( ± 3 days)	Day 43 ( ± 3 days)	Day 56 ( ± 7 days)	Day 73 ( ± 14 days)	CRT day 22 ( ± 3 days)	3 months post CRT	6, 12, 18, 24, 30, and 36 months post CRT
Informed consent	X								
Demographic data^3^/Medical history	X								
Physical examination^4^	X	X	X	X		X	X	X	
Hematology^5^	X	X	X	X		X	X	X	
Chemistry^6^	X	X	X	X		X	X	X	
βHCG pregnancy test^7^	X					X			
Serology^8^	X								
CT chest/abdomen	X^9^				X			X	X
mpMRI of the bladder	X				X			X^10^	
Cystoscopy	X^11^				X			X	X
Concomitant medication^12^	X	X	X	X		X	X	X	
ECG	X								
Adverse events^13^		X	X	X	X	X	X	X	X^14^
Plasma (ctDNA)		X	X	X	X			X	X^15^
Germline DNA (blood)		X							
Urine (ctDNA)		X			X			X	X^15^
Tumor tissue collection	X^16^				X^17^				
HR-QoL & bladder function^18^		X			X			X	X^19^

^1^ Chemotherapy contains mitomycine C on day 1 (12 mg/m^2^) and either oral capecitabin 825 mg/m^2^ twice daily on each day radiotherapy is given, or 5-fluorocuracil 500 mg/m^2^ IV on day 1-5 and day 22-26. ^2^ After 36 months, patients will continue follow-up according to local guidelines. Patients will be contacted every 6 months to record disease recurrence, new cancer treatments and survival. ^3^ Age, gender, smoking status. ^4^ WHO performance status, weight, height (baseline only), temperature, pulse, blood pressure, respiratory rate. Complete examination only at baseline, focused exam at other time points.^5^ Hb, white blood cell differentiation and platelet count, Hct. ^6^ LDH, phosphorus, sodium, potassium, magnesium, chloride, calcium, creatinine, albumin, AST, ALT, bilirubin (ind + dir), GGT, alkaline phosphatase, glucose, lipase, amylase, TSH, fT4, ACTH, cortisol, CRP, ESR. ^7^ Only WOCBP. Negative pregnancy test result should be available within 2 weeks prior to drug administration (day 1, cycle 1). ^8^ HbsAg, HCV RNA. ^9^ Should not be older than 6 weeks at the time of study registration. ^10^ Response evaluation post-CRT will be done in selected centers. ^11^ Repeat only if not done in diagnostic work-up <8 weeks before enrollment. ^12^ Start and stop date should be registered, dosage is only required for immunosuppressive agents. ^13^ Adverse events will be graded according to CTCAE 5.0, SAE reported up to 100 days post last infusion. ^14^ During follow-up, only treatment-related AEs will be monitored. ^15^ Until 12 months after completion of CRT. ^16^ TUR-B does not have to be repeated during screening, but TUR-B tissue has to be collected during screening. ^17^ In case of treatment failure, a TUR-B will be performed and a patient may either proceed to CRT or switch to standard therapy (e.g. chemotherapy and/or cystectomy). ^18^ By Questionnaires: EORTC QLQ-C30 and QLQ - BLM30. ^19^ Until 12 months after completion of CRT.

ipi, Ipilimumab; nivo, nivolumab; CRT, chemoradiation; mpMRI, multiparametric magnetic resonance imaging; ECG, electrocardiogram; ctDNA, circulating tumor deoxyribonucleic acid; HR-QoL, quality of life; IV, intravenous; WHO, world health organization; WOCBP, women of childbearing potential; HBsAg, Hepatitis B surface antigen; HCV RNA, Hepatitis C ribonucleic acid; CTCAE, common terminology criteria for adverse events; SAE, serious adverse event; TUR-B, transurethral resection of bladder tumor; EORTC, European organization for research and treatment of cancer; QLQ-C30, general health-related quality of life questionnaire; QLQ-BLM30, bladder cancer-specific health-related quality of life questionnaire.

### 2.4 Endpoints

#### 2.4.1 Primary endpoint

The primary objective of the INDIBLADE trial is to establish efficacy of induction therapy with ipilimumab plus nivolumab followed by chemoradiation by determining BI-EFS, as this is a clinically meaningful endpoint for the population included in this trial. Events are defined as muscle-invasive recurrence in the bladder or in the ureter - distal of the crossing with the common iliac artery -, nodal or distant recurrence, cystectomy, death by any cause and/or switch to platinum-based chemotherapy. If possible, histological or cytological confirmation of disease recurrence is preferred. BI-EFS will be determined starting from initiation of the study drug with the use of cystoscopy and CT-imaging of the chest and abdomen. We expect the patient population in INDIBLADE to consist of approximately 40% cT2N0 tumors, 40% cT3-T4aN0 tumors, and 20% patients with node positive disease, which represents a population with a poor prognosis. This is underlined by results of a large retrospective study in which patients with mainly cT2 tumors were evaluated after either cystectomy or chemoradiation, demonstrating a median OS of 3 years and 2.7 years respectively (42). In addition, in a study evaluating different neo-adjuvant chemotherapy regimens in node negative patients with mainly cT2 tumors, PFS after three years ranged between 56-66% for different treatment regimens, thereby emphasizing the poor prognosis of patients with cT2 tumors (43). We therefore aim to exclude a lower level of BI-EFS of 50% at two years, which corresponds to a median BI-EFS of 24 months. Based on efficacy of the ICB regimen in NABUCCO (pCR ipi high cohorts 46% and 43%) (26, 27), we aim to achieve a BI-EFS of 70% at two years in the current trial, corresponding to a median BI-EFS of 46.6 months. To accomplish this result, using a two-sided one-sample log-rank test with a power of 81.32% and a 5% significance level, and considering an accrual period of 30 months and follow-up period of a minimum of 12 months after registration of the last study participant, 50 patients need to be recruited.

#### 2.4.2 Secondary endpoints

##### 2.4.2.1 Overall- and progression-free survival

Secondary objectives to evaluate efficacy include OS and PFS. OS will be measured from patient enrollment until death. If information about survival is lacking, OS will be censored on the last date the patient was known to be alive. PFS will be measured from initiation of the study drug until one of the following events: muscle-invasive recurrence in the bladder or in the ureter - distal of the crossing with the common iliac artery, nodal or distant recurrence, switch to platinum-based chemotherapy or death by any cause. Performing a cystectomy will not be considered as a PFS event as PFS is meant to determine efficacy of induction therapy with ICB, regardless of loco-regional treatment. We will report on OS and PFS after two and five years.

##### 2.4.2.2 Safety

As treatment-related adverse events (AEs) potentially influence (health-related quality of life (HR-QoL) related) outcome, we will evaluate safety at various time points within this trial: during and after induction therapy with ICB and during chemoradiation. AEs of all grades irrespective if related to treatment will be provided as measured according to CTCAE 5.0 until 100 days after study drug initiation. Once this period of 100 days after study drug initiation has expired, only treatment-related AEs will be registered.

##### 2.4.2.3 Predictive value of mpMRI

The evaluation of neo-adjuvant treatment response in (locally advanced) bladder cancer, using conventional imaging, remains challenging. This is partially due to the changes after TUR-B, including an inflammatory response. MpMRI is hypothesized to more accurately distinguish TUR-B effects from cancer (recurrence) (44, 45). Anatomical T2 weighted (T2W) images have superior soft tissue contrast as compared to CT images. Additionally, anatomical information is combined with functional information from the diffusion weighted images and dynamic contrast enhanced images. Based on encouraging results of mpMRI in the Pure-01 trial (34) and our own results on mpMRIs from NABUCCO, we will perform an mpMRI at baseline (after TUR-B), after finalizing ICB treatment, and after completing chemoradiation to assess its potential to predict response. As there is a delay of at least several weeks between TUR-B and recruitment in the current trial, we expect that inflammatory effects that were caused by TUR-B will have subsided at the time of the mpMRI of the bladder at baseline. To enhance predictive accuracy and reduce interobserver variability during image analysis, we will use an AI-based algorithm to identify non-responding patients who might benefit from a different treatment regimen.

##### 2.4.2.4 Clearance of ctDNA

Current assays to measure plasma ctDNA use highly sensitive sequencing methods to detect in a set of mutations in plasma that is present in a patient’s tumor. Clearance of ctDNA, as measured by these assays, has shown striking predictive power for clinical outcome after treatment with neo-adjuvant or adjuvant atezolizumab in resectable bladder cancer (46, 47). Based on these encouraging results, clearance of plasma ctDNA following treatment with pre-operative ipilimumab plus nivolumab was investigated in NABUCCO (27). Results showed a strong correlation between plasma ctDNA clearance before surgery and both treatment response (odds ratio 45.0) and PFS (hazard ratio 10.4), which suggests that absence of ctDNA predicts for pCR and could potentially select bladder cancer patients for de-escalation of locoregional therapy, for example by using a bladder-sparing approach. In the current study, we will collect liquid biopsies to evaluate the presence of ctDNA. We will draw blood to obtain plasma at baseline, before each ICB cycle, at both response evaluations, and during follow-up until 12 months after completing chemoradiation. Urine will be collected at baseline, at both response evaluations and after six and twelve months after completing chemoradiation.

##### 2.4.2.5 Health-related Quality of Life

Data regarding HR-QoL upon treatment for MIBC are scarce, in particular due to short follow-up periods and the lack of sufficient prospective studies. Nevertheless, results of two prospective trials demonstrate that radical cystectomy can result in long-term effects on HR-QoL, with sexual dysfunction being a main domain compromised by surgery (48, 49). Bladder-sparing treatment on the other hand resulted in a short-term drop directly after chemoradiation, followed by a long-term HR-QoL comparable to baseline levels (50). To learn more about HR-QoL and bladder function following the bladder-sparing treatment regimen provided in the INDIBLADE trial, we prospectively collect questionnaires regarding QoL (QLQ30) and bladder function (BLM30) provided by the European Organisation for Research and Treatment of Cancer (EORTC) at baseline, at each response evaluation, and during follow-up until 12 months after finalizing chemoradiation.

### 2.5 Patient participation

To achieve successful patient recruitment and adherence to the study protocol, patient participation is of vital importance. We installed a patient advisory committee at an early stage of the development of this trial to ensure that both the trial conduct and outcome measurements are comparable to patients’ treatment goals. Also throughout patient enrollment in this trial, we will continue to involve the opinion of representatives of the patients’ association by scheduling yearly meetings to optimize patient participation.

### 2.6 Ethics and dissemination

The INDIBLADE trial obtained authorization by the medical ethical committee of the Netherlands Cancer Institute (NKI) in Amsterdam and by the Central Committee on Research Involving Human Subjects. Additionally, the medical ethical committee of all participating sites reviewed the study protocol before initiation of the study site. In case of a substantial amendment to the protocol, a review will be performed by the medical ethical committee of the NKI before integrating the amended protocol. According to the Declaration of Helsinki, all participants give written informed consent before enrollment in the study. The INDIBLADE trial is registered at clinicaltrials.gov (NCT05200988). At the NKI, patient enrollment started in February 2022. By May 2023, 31 patients have been recruited.

## 3 Discussion

Here, we provide a description of the clinical protocol of the INDIBLADE trial. To our knowledge, this is the first clinical trial in which ICB is administrated as induction therapy followed by consolidating chemoradiation to patients with (locally advanced) muscle-invasive bladder cancer to evaluate BI-EFS. There are several ongoing trials assessing bladder-preserving strategies using ICB and chemoradiation, but in contrast to the current trial, these trials simultaneously administer ICB and chemoradiation. In a phase 2 trial evaluating bladder-intact disease-free survival at two years, patients with cT2-T4a MIBC are treated with pembrolizumab followed by TMT with gemcitabine as radio sensitizing agent together with pembrolizumab (51). In another phase 2 study, patients with cT2-T4a MIBC are treated with radiation, cisplatin as radio sensitizing chemotherapy, and pembrolizumab concurrently to evaluate feasibility of this regimen (52). In the ongoing phase 2 CRIMI trial, efficacy of concurrent chemoradiation combined with nivolumab or with ipilimumab 1 mg/kg plus nivolumab 3 mg/kg in patients with T2-4aN0-1 bladder cancer is being evaluated (53).

A biological argument for sequential administration of ICB and chemoradiation includes the immunosuppressive effects of radiation on the TME, which could diminish the immune response evoked by ICB (54). However, we do consider the use of ICB and chemoradiation attractive because of their different mechanisms of action. We therefore opt for sequential use in order to circumvent the negative effects on the TME, while preserving the high efficacy of ICB in the “neo-adjuvant” setting. Whereas the immunological effects of chemotherapy on the urothelial TME are still largely unknown, studies using combinations of chemotherapy and ICB simultaneously have shown disappointing results. The IMvigor 130 trial, a phase 3 study evaluating PFS and OS in metastatic urothelial cancer patients treated with chemotherapy plus simultaneous atezolizumab or placebo did not reach the prespecified level of significance with respect to OS, suggesting that concurrent use of anti-PD-L1 and chemotherapy does not result in superior OS compared to chemotherapy alone (15). Similar results were obtained in the KEYNOTE 361, showing no additional benefit of combining pembrolizumab and chemotherapy simultaneously versus chemotherapy alone as first line treatment in metastatic urothelial cancer (12).

Conversely, sequential administration of chemotherapy and ICB has shown positive results. In a phase 3 trial evaluating maintenance avelumab in patients with metastatic urothelial cancer who did not progress on first line chemotherapy, avelumab prolonged OS, compared to best supportive care (10). The phase 3 CHECKMATE-274 trial investigating adjuvant nivolumab showed that patients who received neo-adjuvant chemotherapy had a more pronounced disease-free survival benefit from adjuvant nivolumab compared to patients who underwent upfront cystectomy without neo-adjuvant chemotherapy (55).

In the current trial, we initially treat patients with ICB as induction treatment before continuing with chemoradiation. As was observed in the NABUCCO trial, we expect tumor volume to shrink upon ICB, thereby enabling omission of cystectomy, including in patients with high-risk disease. In addition, we hypothesize that as the primary tumor has not been removed, the presence of multiple neo-antigens elicits a broader immune response (56, 57). Once the tumor has decreased in volume, the goal of chemoradiation is to combat residual tumor cells. Several ongoing trials investigate the reverse sequential approach compared to INDIBLADE, employing chemoradiation followed by ICB. In a phase 2 trial, patients with stage II-IV bladder cancer are treated with nivolumab within 90 days after completing standard of care chemoradiation to assess failure-free survival at two years (58). The BladderSpar trial evaluates disease-free survival with sequential administration of chemoradiation followed by atezolizumab in patients with cT2-3N0 bladder tumors (59). Final results have to be awaited before drawing any conclusion.

In conclusion, we here provide the design and rationale for the INDIBLADE trial, which is the first clinical trial in patients with cT2-4aN0-2 bladder cancer evaluating BI-EFS upon induction therapy with combination ICB and subsequent consolidative chemoradiation. Results of this trial will not only provide information about the efficacy of ICB and chemoradiation as a bladder-preservation strategy, but will also give insight into the potential of ctDNA assessment and mpMRI of the bladder to identify patients who qualify for a bladder-sparing approach and those who may need alternative systemic therapy. Furthermore, HR-QoL assessment of our bladder-sparing approach is a vital component of this trial.

## Data availability statement

The original contributions presented in the study are included in the article/supplementary material. Further inquiries can be directed to the corresponding author.

## Ethics statement

The studies involving humans were approved by Medical Ethical Committee of the Netherlands Cancer Institute and the Central Committee on Research Involving Human Subjects. The studies were conducted in accordance with the local legislation and institutional requirements. The participants provided their written informed consent to participate in this study.

## Author contributions

MH, ES, BR, BS, RM, DR, JB, MF, SB, TB and J. Nooteboom (radiotherapist University Medical Center Utrecht, The Netherlands) contributed to the design of the INDIBLADE study. CS and MH wrote the current manuscript. All co-authors reviewed and approved the manuscript before submission.
